# Football and team handball training postpone cellular aging in women

**DOI:** 10.1038/s41598-021-91255-7

**Published:** 2021-06-03

**Authors:** Marie Hagman, Bjørn Fristrup, Rémi Michelin, Peter Krustrup, Muhammad Asghar

**Affiliations:** 1 Department of Sports Science and Clinical Biomechanics, SDU Sport and Health Sciences Cluster (SHSC), Faculty of Health Sciences, University of Southern Denmark, Campusvej 55, 5230 Odense, Denmark; 2 Institute of Sports Medicine Copenhagen, Bispebjerg Hospital, 2400 Copenhagen, NV Denmark; 3 Division of Infectious Diseases, Department of Medicine Solna, Karolinska Institutet, 171 64 Solna, Sweden; 4 Danish Institute for Advanced Study (DIAS), University of Southern Denmark, Odense, Denmark; 5 Sport and Health Sciences, St Luke’s Campus, University of Exeter, Exeter, EX1 6JA UK; 6 Shanghai University of Sport, Shanghai, China; 7 Department of Infectious Diseases, Karolinska University Hospital, Solna, Sweden

**Keywords:** Cell biology, Molecular biology, Physiology, Biomarkers, Diseases, Risk factors

## Abstract

Several hallmarks of aging have been identified and examined separately in previous exercise studies. For the first time, this study investigates the effect of lifelong regular exercise in humans on two of the central aging hallmarks combined. This cross-sectional study involved 129 healthy, non-smoking women, including young elite football players (YF, *n* = 29), young untrained controls (YC, *n* = 30), elderly team handball players (EH, *n* = 35) and elderly untrained controls (EC, *n* = 35). From a resting blood sample, mononuclear cells (MNCs) were isolated and sorted into monocytes and lymphocytes. Telomere length, mitochondrial (mtDNA) copy number and key regulators of mitochondrial biogenesis and function (PGC-1α and PGC-1β expression) were measured using quantitative polymerase chain reaction (qPCR). Overall, young women showed significantly longer telomeres and higher PGC-1α and PGC-1β expression, but lower mtDNA copy number compared to elderly subjects. A multivariate analysis showed that YF had 22–24% longer telomeres in lymphocytes and MNCs compared to YC. In addition, YF showed 19–20% higher mtDNA copy number in lymphocytes and MNCs compared to YC. The two young groups did not differ in PGC-1α and PGC-1β expression. EH showed 14% lower mtDNA copy number in lymphocytes compared to EC, but 3.4-fold higher lymphocyte PGC-1α expression compared to EC. In MNCs, EH also showed 1.4–1.6-fold higher PGC-1α and PGC-1β expression. The two elderly groups did not differ in telomere length. Elite football training and lifelong team handball training are associated with anti-aging mechanisms in leukocytes in women, including maintenance of telomere length and superior mitochondrial characteristics.

## Introduction

In a time of consistent and considerable increase in global life expectancy^1^, healthy aging and improved quality of life in old age are a major challenge. Aging is defined as time-dependent deterioration of the body with the passage of time that increase vulnerability to death. One of the central hallmarks of aging is telomere attrition^2^. Telomeres, which are specific nucleoprotein structures capping both ends of each chromosome, function to maintain genome stability and preserve genetic information. As we age, human telomeres gradually shorten due to successive cell division and incomplete replication. When telomeres reach a critical length, the cell can no longer divide and become senescent^3^. Accelerated telomere attrition has been proposed as a risk factor for several human pathologies and age-related diseases, such as chronic inflammation, infection, dementia, diabetes, cardiovascular diseases (CVD) and cancer, and for mortality in general^4,5^. By contrast, longer telomeres have been shown to be positively associated with more years of healthy living^6^. Another important mechanism for extending both health and lifespan is maintenance of mitochondrial function. Indeed, decreased mitochondrial function, which results in impaired adenosine triphosphate (ATP) generation and increased levels of reactive oxygen species (ROS), has been implicated in driving the aging process^7^. It has been found that telomere shortening and associated DNA damage promote mitochondrial dysfunction, diminished oxidative defence and compromised energy-generating processes^8^. Thus, regulation of telomeres and mitochondria may be directly linked in the process of aging and in age-associated disease development.

Previous studies show that engagement in physical activity is associated with healthy aging and decreased risk of chronic diseases^9^, whereas the relationship between telomere length and level of physical training is still a matter of some debate. A systematic review from 2020 showed that better cardiorespiratory fitness or a large cardiorespiratory training load are associated with longer telomeres in older healthy humans, but not in young subjects^10^. This observation is in line with the hypothesis that telomere length is stable in young age, but begins to decline in older adulthood^11^. Inconsistent findings regarding the role of exercise in telomere shortening may also be related to exercise modality. Werner et al.^12^ have recently shown that 6 months of either endurance training or interval training, but not resistance training, can increase telomere length in previously inactive adults. Aerobic exercise training is also considered the gold standard for improving mitochondrial biogenesis in all age groups. In older adults, aerobe exercise training may partially reverse mitochondrial dysfunction by increasing the mitochondrial (mtDNA) copy number and volume, mitochondrial transcript and protein expression, ATP synthesis and oxidative enzyme function, while the effect of resistance training on mitochondrial function is less certain^13^.

The type of exercise also appears to have a high impact on the motivation to maintain lifelong attendance. Being part of a community and developing relationships are two of the main reasons why older adults keep participating in sports^14^. Team sports, such as football and team handball, are characterised by an important social factor, while combining endurance, interval and resistance training in one activity^15,16^. Hence, in a global strategy to increase physical activity, team sports may offer unique qualities. Whereas the topic of “Football for Health” has received much attention, with more than 150 scientific publications during the last 15 years^17^, most research studies within the field of team handball have focused on injuries and performance in elite players. However, the concept of team handball training as a health-promoting activity has slowly gained interest within the last couple of years. Indeed, positive cardiovascular, skeletal and muscular adaptations have recently been observed following a short period of recreational team handball training^18,19^. To the best of our knowledge, no studies have investigated the potential cellular anti-aging effects of either team handball or elite football training in women. Thus, the aim of the present cross-sectional study was to examine telomere length, mtDNA copy number and key regulators of mitochondrial biogenesis and function (PGC-1α and PGC-1β) in elderly female team handball players and young female elite football players in comparison with age-matched untrained women.

## Results

### Telomere length

**Figure 1:**
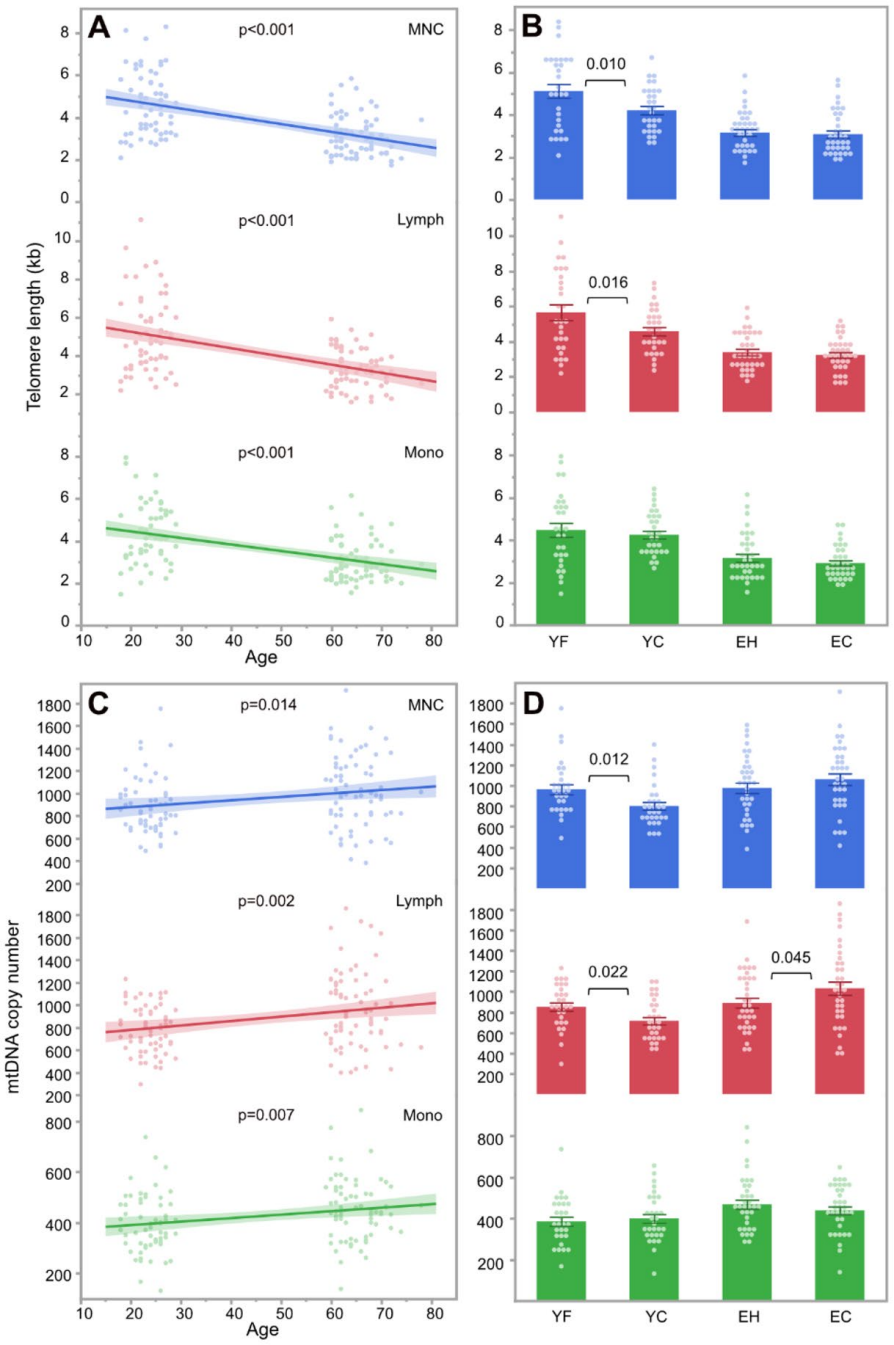
Correlation of telomere length with age **(A)** and group **(B)** as well as mtDNA copy number according to age **(C)** and group **(D)** in young football players (YF), young controls (YC), elderly team handball players (EH) and elderly controls (EC). *Lymph* lymphocytes; *MNC* mononuclear cells; *Mono* monocytes.

In total, 129 subjects were included in all the analyses. Overall, telomere length was negatively correlated with age in all cell types, including lymphocytes (r^2^ = 0.26, p < 0.001), monocytes (r^2^ = 0.23, p < 0.001) and MNCs (r^2^ = 0.28, p < 0.001), with young subjects showing 44–54% longer telomeres than elderly subjects depending on the cell type (Fig. 1A). Our multivariate analysis corrected for age showed that young football players (YF) had 24% longer telomeres in lymphocytes compared to young controls (YC) (5.67 ± 0.45 vs 4.59 ± 0.24 kb, p = 0.016) and 22% longer telomeres in MNCs compared to YC (5.12 ± 0.33 vs 4.21 ± 0.19 kb, p = 0.010, Fig. 1B). The elderly team handball players (EH) and elderly controls (EC) did not differ in telomere length in any cell types (all p > 0.05, Fig. 1B, Suppl. Table S1).

### Mitochondrial copy number and PGC-1α and PGC-1β expression

**Figure 2:**
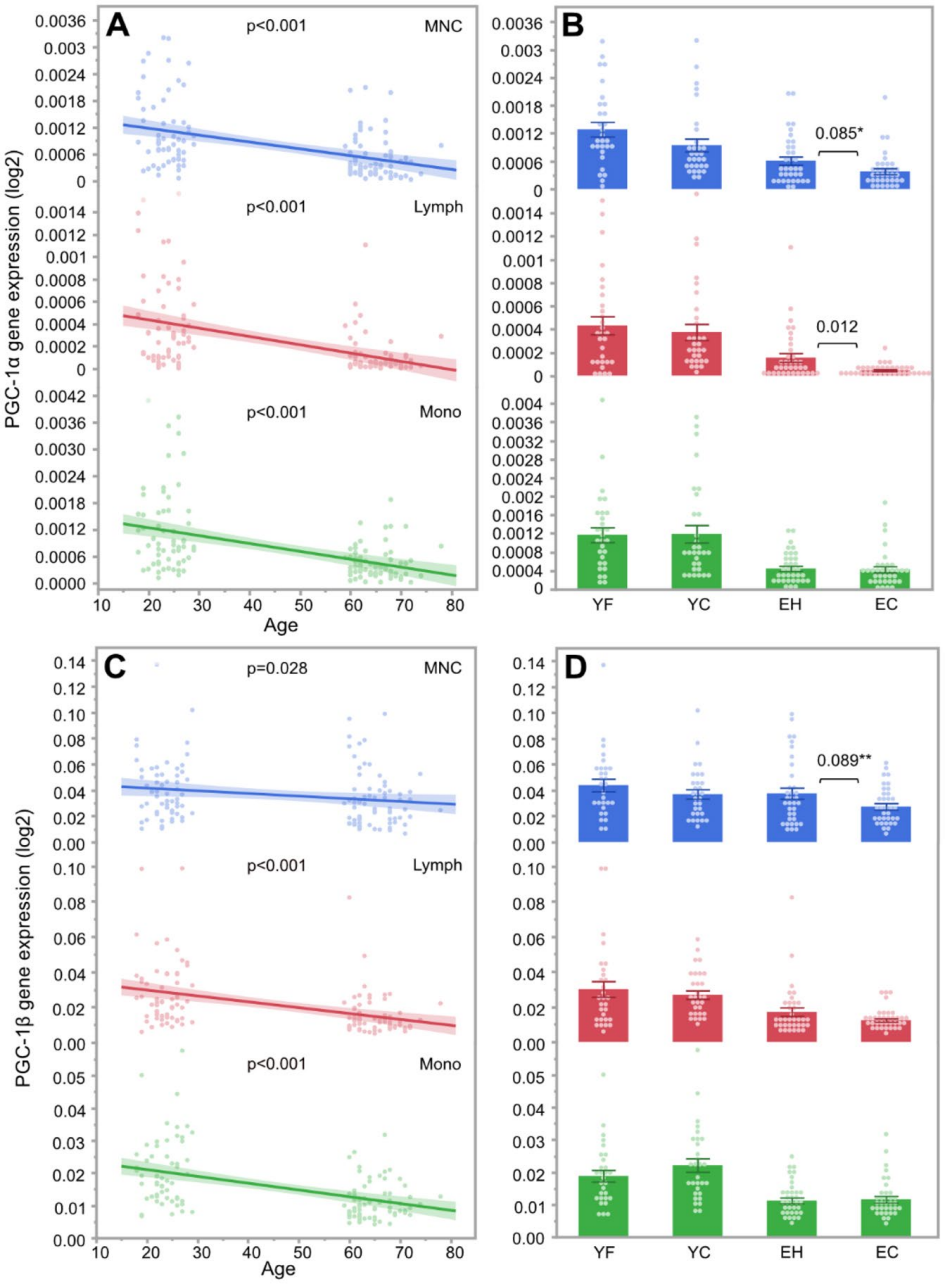
Correlation of PGC-1α gene expression with age **(A)** and group **(B)** as well as PGC-1β gene expression according to age **(C)** and group **(D)** in young football players (YF), young controls (YC), elderly team handball players (EH) and elderly controls (EC). *Lymph* lymphocytes; *MNC* mononuclear cells; *Mono* monocytes. *p = 0.041 in univariate analysis; **p = 0.044 in univariate analysis.

Overall, the mtDNA copy number was positively correlated with age in all cell types (lymphocytes: r^2^ = 0.07, p = 0.002; monocytes: r^2^ = 0.06, p = 0.007; MNCs: r^2^ = 0.05, p = 0.014), with elderly participants showing a 16–23% higher mtDNA copy number than young participants depending on the cell type (Fig. 1C). On the other hand, PGC-1α and PGC-1β expression was negatively correlated with age in lymphocytes (PGC-1α: r^2^ = 0.22, p < 0.001; PGC-1β: r^2^ = 0.17, p < 0.001), monocytes (PGC-1α: r^2^ = 0.22, p < 0.001; PGC-1β: r^2^ = 0.22, p < 0.001) and MNCs (PGC-1α: r^2^ = 0.20, p < 0.001; PGC-1β: r^2^ = 0.04, p = 0.028), with young participants showing a 1.2–4.0-fold higher genes expression compared to elderly participants depending on the cell type (Fig. 2A,C).

Comparison of the two young groups using multivariate analysis showed 19% higher lymphocyte mtDNA copy number in YF compared to YC (853 ± 39 vs 715 ± 36, p = 0.022) as well as 20% higher MNC mtDNA copy number in YF compared to YC (962 ± 49 vs 801 ± 38, p = 0.012, Fig. 1D, Suppl. Table S2). The expression of PGC-1α and PGC-1β did not differ between YF and YC in any cell types (all p > 0.05, Fig. 2B,D, Suppl. Table S3).

In the multivariate analysis, EH showed a 14% lower mtDNA copy number in lymphocytes compared to EC (891 ± 48 vs 1032 ± 65, p = 0.045, Fig. 1D), while the PGC-1α expression was 3.4-fold higher in the lymphocytes in EH compared to EC (p = 0.012, Fig. 2B). In a univariate analysis, EH also showed 1.6-fold higher PGC-1α expression in MNCs compared to EC (p = 0.041, Fig. 2B) as well as 1.4-fold higher MNC PGC-1β expression compared to EC (p = 0.044, Fig. 2D). However, in the multivariate analysis the differences between EH and EC in PGC-1α and PGC-1β expression in MNCs only tended to be significant (PGC-1α: p = 0.085, PGC-1β: p = 0.089, Suppl. Table S3).

### Body composition and VO_2max_

Regular team handball and football training were associated with profound improvements in body composition and VO_2max_ (Table 1). Compared to YC, YF showed 22% lower total body fat percentage (p < 0.001), 32% lower android fat percentage (p < 0.001), 20% lower gynoid fat percentage (p < 0.001), 15% lower android/gynoid (A/G) fat ratio (p = 0.017), 5.8 kg higher total lean mass (p < 0.001), 2.3 kg higher leg lean mass (p < 0.001) and 27% higher VO_2max_ (p < 0.001, Table 1). Compared to EC, EH showed 16% lower total body fat percentage (p < 0.001), 22% lower android fat percentage (p = 0.002), 14% lower gynoid fat percentage (p < 0.001), 3.6 kg higher total lean mass (p < 0.001), 1.2 kg higher leg lean mass (p = 0.005) and 31% higher VO_2max_ (p < 0.001, Table 1). Total lean mass was even 3.0 kg higher in EH compared to young controls (YC) (p = 0.011).

**Table 1:** Group characteristics in young football players (YF), young controls (YC), elderly team handball players (EH) and elderly controls (EC).

	YF (*n* = 29)				YC (*n* = 30)				EH (*n* = 35)				EC (*n* = 35)			
Age (years)	22.5 ± 0.6				24.9 ± 0.4^§§^				63.9 ± 0.7				66.1 ± 0.6^†^			
**Exercise/fitness**																
All exercise (h/week)	9.0 ± 0.4^###^				0.1 ± 0.0				4.7 ± 0.5***				0.2 ± 0.1			
Team handball/football (h/week)	6.7 ± 0.2				–				2.0 ± 0.1				–			
VO_2max_ (ml/min/kg)	45.3 ± 1.0^###^				35.7 ± 0.9				30.2 ± 1.2***				23.1 ± 0.8			
**Body composition**																
Body mass (kg)	65.8 ± 1.6				64.3 ± 2.3				69.2 ± 1.5				70.5 ± 1.9			
Total body fat (%)	26.3 ± 0.8				33.5 ± 1.1^§§§^				34.4 ± 1.4				41.1 ± 1.0^†††^			
Android fat (%)	20.8 ± 1.2				30.6 ± 2.0^§§§^				34.6 ± 2.3				44.1 ± 1.8^††^			
Gynoid fat (%)	30.7 ± 0.9				38.3 ± 0.9^§§§^				37.2 ± 1.2				43.3 ± 0.8^†††^			
A/G ratio	0.7 ± 0.0				0.8 ± 0.0^§^				0.9 ± 0.0				1.0 ± 0.0			
Total lean mass (kg)	46.3 ± 0.9^###^				40.5 ± 1.0				43.4 ± 0.6***				39.8 ± 0.7			
Leg lean mass (kg)	16.7 ± 0.4^###^				14.4 ± 0.4				15.0 ± 0.3**				13.8 ± 0.3			
**Telomere length (kb)**																
MNC	5.12 ± 0.33^#^				4.21 ± 0.19				3.16 ± 0.16				3.08 ± 0.17			
Lymphocytes	5.67 ± 0.45^#^				4.59 ± 0.24				3.40 ± 0.18				3.25 ± 0.16			
Monocytes	4.47 ± 0.33				4.25 ± 0.18				3.16 ± 0.19				2.91 ± 0.13			
**mtDNA copy number**																
MNC	962 ± 49^#^				801 ± 38				976 ± 51				1061 ± 56			
Lymphocytes	853 ± 39^#^				715 ± 36				891 ± 48				1032 ± 65^†^			
Monocytes	386 ± 22				399 ± 21				468 ± 22				439 ± 19			

Group means ± SEM. ^#^p < 0.05, ^###^p < 0.001, higher than YC; ^§^p < 0.05, ^§§^p < 0.01, ^§§§^p < 0.001, higher than YF; **p < 0.01, ***p < 0.001, higher than EC; ^†^p < 0.05, ^††^p < 0.01, ^†††^p < 0.001 higher than EH.

*A/G ratio* android/gynoid fat ratio; *MNC* mononuclear cells; *VO2max* maximal oxygen consumption.

### Correlations between cellular aging markers, exercise variables and body composition

**Figure 3:**
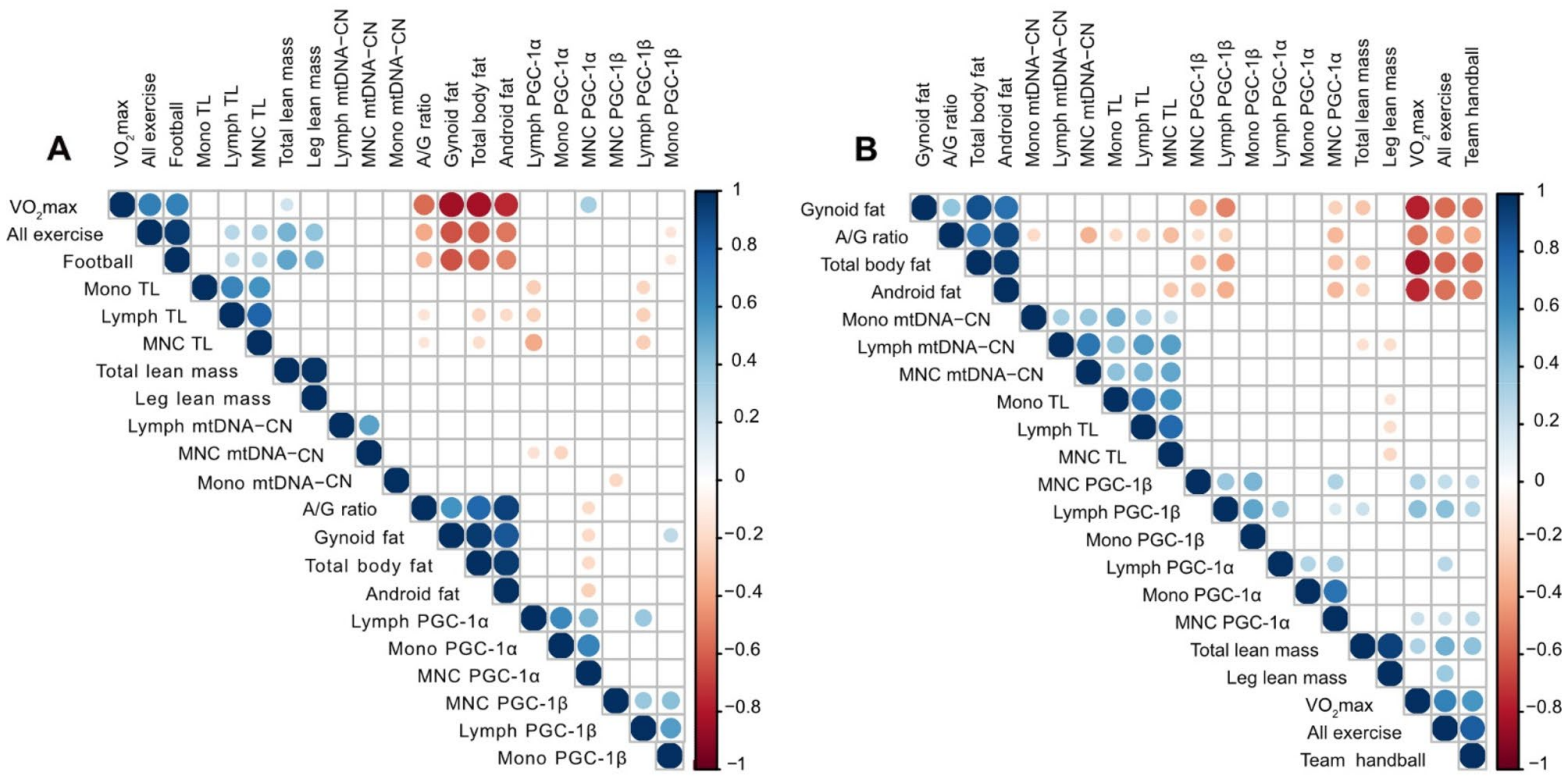
Spearman’s correlations between the investigated variables visualised as arc diagrams in young (18‒30 years) women (*n* = 59) **(A)** and elderly (60‒80 years) women (*n* = 70) **(B)**. *A/G ratio* android/gynoid fat ratio; *All exercise* hours of weekly exercise of all types; *Football* hours of weekly football training; *Lymph* lymphocytes; *MNC* mononuclear cells; *Mono* monocytes; *mtDNA-CN* mitochondrial copy number; *Team handball* hours of weekly team handball training; *TL* telomere length; *VO*_*2*_*max* maximal oxygen consumption.

Spearman’s correlations were made for all variables in young and elderly women separately. Among the young women, the total amount of weekly exercise and the amount football training were positively correlated with telomere length in lymphocytes and MNCs, while VO_2max_ was positively correlated with MNC PGC-1α expression (all p < 0.05, Fig. 3A, Suppl. Table S4). Among the elderly women, VO_2max_, the total amount of weekly exercise and team handball training were all positively correlated with PGC-1α and PGC-1β expression in MNCs and PGC-1β expression in lymphocytes (all p < 0.05, Fig. 3B, Suppl. Table S5). By contrast, body fat percentage and A/G ratio were negatively correlated with PGC-1α and PGC-1β expression in MNCs and PGC-1β expression in lymphocytes in the elderly women (all p < 0.05, Fig. 3B, Suppl. Table S5). Finally, telomere length and mtDNA copy number were positively correlated in all cell types in the elderly participants only (all p < 0.05, Fig. 3B, Suppl. Table S5).

## Discussion

This cross-sectional study is the first to investigate the effect of football and team handball training on telomere length and mitochondria in women. Furthermore, inclusion in the same study of two essential hallmarks of aging is quite unique and may lead to a broader understanding of the potential anti-aging effects of regular exercise. The main findings were that: (1) young elite football players (YF) had longer telomeres in both lymphocytes (24%) and MNCs (22%) compared to untrained young controls (YC) as well as a higher mtDNA copy number in lymphocytes (19%) and MNCs (20%) compared to YC; and (2), lifelong trained elderly team handball players (EH) had a lower mtDNA copy number in lymphocytes (14%) compared to untrained elderly controls (EC), but higher PGC-1α expression in lymphocytes (3.4-fold) compared to EC. PGC-1α and PGC-1β expression in MNCs was also higher (~ 1.5-fold) in EH compared to EC according to the univariate analysis.

Telomere length has been described as a ‘biological clock’, which can be used not only to establish the biological age of an individual, but also to estimate the risk of age-related diseases^4^. Telomere attrition is a consequence of normal aging in humans, and it has been established that telomeres gradually shorten with age as a result of the end-replication problem, albeit large intraindividual variation exists^2^. The present study showed that young women on average had 1.55 kb longer telomeres compared to elderly women, which is comparable to differences in telomere length of 1.35 kb previously found between young (18–32 years) and older (55–72 years) sedentary adults of both sexes^20^. With an age difference of 41.4 years between the young and elderly groups in the present study, this difference in telomere length corresponds to a loss of ~ 37 bp per year of chronological age.

Our finding of reduced telomere shortening in young, but not elderly, ball game players contradict previous observations showing an effect of exercise on telomere length in adults above 50 years only. Surprisingly, the 22–24% longer telomeres demonstrated in YF is comparable to beneficial adaptations observed in middle-aged or older athletes. Indeed, experienced ultra-distance runners aged ~ 45 years have shown 25% longer telomeres than sedentary peers^21^, while older (> 65 years) endurance-trained adults have shown 22% longer telomere length compared with older people with medium activity levels^22^. Despite sports participation at elite level and large exercise volumes, changes in telomere length have usually not been detected in young well-trained endurance athletes^20,21,23^, nor in young male elite football players^24^. The inconsistency in observations between young and older adults are most likely explained by the postulation that telomere length and attrition are relatively stable from childhood to adulthood^11^. Thus, the observed findings in YF are quite striking due to the young age of the group (~ 22 years). So far, intervention studies regarding exercise and telomere shortening are sparse. While some studies have not been able to detect changes in telomere length following short-term exercise interventions^25,26^, a few studies have observed minor adaptations of 2–4%^12,27^. Hence, the 22–24% superior telomere length observed in YF was assumed to be a result of many years of regular exercise, here among elite football training. To the best of our knowledge, the effect of football training on telomere shortening has never been investigated. Our findings show that football training at elite level might be particularly effective for achieving cellular anti-aging adaptations in young women.

Our correlation analysis showed that the weekly amount of exercise of all modalities and football training specifically were positively correlated with telomere length in young, but not elderly, women. An association between better cardiorespiratory fitness or a large training load and longer telomeres was found in 80% of all studies included in a recent systematic review^10^. In contrast to our findings, this association was mainly observed in middle-aged and older people, presumably due to the limited telomere attrition in young age. However, at least two studies have found an inverted “U” correlation, showing that both low and very high physical activity are associated with increased telomere shortening^28,29^. Thus, there may be an upper limit of exercise volume, where too much exercise elicits a negative effect on cellular aging. In the present study, 7 h of weekly football training had a positive effect on telomere length, indicating that this amount of football training is not excessive.

A potential explanation for the similar telomere length in EH and EC may be related to the sex and age of the groups. It has been demonstrated that the oestrogen level is positively associated with telomere length, possibly due to the ability of the hormone to upregulate telomerase and concurrently reduce oxidative stress^30^. As EH consisted of postmenopausal women, their oestrogen level was expected to be negligible, and they may not have the capacity to upregulate telomerase activity to the same extent as young women. Although telomere length did not significantly differ between the two elderly groups, the absolute values were 3–8% higher in EH compared to EC depending on the cell type. In a recent male football study by Hagman et al.^24^, differences in telomere length of only 1.3–2.5% were found to be statistically significant using the fluorescence in situ hybridisation coupled with flow cytometry (Flow-FISH) technique, but not with the qPCR method. Previous validation studies have confirmed that Flow-FISH may be more sensitive when measuring telomere length^31^, and we speculate whether use of this technique or a larger sample size would have resulted in significant findings in EH. It is, however, possible that football training is superior to team handball training regarding achievement of telomere adaptations, but further studies are necessary to address this.

Mitochondria are characterised as ‘powerhouses of the cell’, as their primary role is to supply ATP from aerobic respiration for growth, development and preservation of the cell^13^. However, mitochondria also play a central role in the aging process through their vital functions for cell survival, including inflammation, ROS production, senescence, and apoptosis. In the present study, we found a higher mtDNA copy number in elderly women compared to young. This age-related increase in mtDNA copy number might be a consequence of, and compensatory mechanism for, a decline in PGC-1α and PGC-1β expression or an increased accumulation of mtDNA mutations and damage with age which leads to dysfunctional mitochondria and impaired mitochondrial respiration^32^. While PGC-1α is often referred to as the ‘master regulator’ of mitochondrial biogenesis^33^ and mediates adaptations in tissues with high-energy needs, PGC-1β is mainly believed to participate in the maintenance of basal mitochondrial function^34^. Besides being involved in mitochondrial biogenesis, PGC-1α influences several other gene expressions involved in metabolic pathways, and strongly correlates with other markers of mitochondrial function including citrate synthase (CS) in healthy individuals^35^.

While few studies have investigated mitochondria in male football players^36,37^, mitochondrial characteristics has, to the best of our knowledge, never been studied in female elite football players. Furthermore, this is the first study to examine the effects of team handball training on mitochondria. In the present study, YF showed a 19–20% higher mtDNA copy number compared to YC. Meanwhile, EH showed a 14% lower lymphocyte mtDNA copy number compared to EC, but 3.4-fold higher PGC-1α expression in lymphocytes. Interestingly, the mtDNA copy number was comparable in the two exercise groups despite the large age difference, while the two non-exercise groups showed fluctuations in mtDNA copy number. A similar PGC-1α and PGC-1β expression observed in the two young groups was most likely explained by the markedly higher mtDNA copy number in YF. Our results show that an upregulation of the mitochondrial content, was favoured in young athletes, potentially due to sufficiently high mitochondrial function in young age.

Mitochondria are highly plastic organelles that can be remodelled in several ways according to the energetic challenges of the cell. These processes can be triggered by acute and/or chronic physical activity, and mitochondrial adaptations are generally expected following aerobic exercise training^13^. Previous studies indicate that the type of exercise training is decisive for the specific and diverse mitochondrial adaptations^33^. Indeed, a recent review concluded that training volume is crucial for changes in mitochondrial content, while exercise intensity appears to be important for changes in mitochondrial function^38^. Football and team handball training are both characterised as intermittent high-intensity exercise^16,17^. Although the exercise intensity during team handball training has never been investigated in elderly women, Hornstrup et al.^39^ have previously shown that the average heart rate during team handball training is very high (~ 85% of HR_max_) and independent of prior team handball experience. Thus, sufficiently high exercise intensities necessary for changes in mitochondrial biogenesis and function were anticipated in both YF and EH. Increased PGC-1α expression has previously been observed in lifelong trained male football players^37^ and in untrained men following long-term recreational football training^36^, indicating that football training can increase mitochondrial biogenesis in some cases.

The effect of chronic exercise on mitochondrial characteristics is believed to be a cumulative result of repeated bouts of acute exercise, as the expression of PGC-1α has been shown to be markedly and acutely increased by a single session of aerobic exercise^33,40^. As the included participants had not been physically active for minimum 48 h prior to testing, the superior PGC-1α and PGC-1β expression observed in EH was expected to be a cumulative effect of many years of regular team handball training. Although acute changes in PGC-1α and PGC-1β expression have been shown to differ following exercise, a positive correlation between transcription of the two genes has been found^40^. This is in line with the results from the present study showing some positive correlations between PGC-1α and PGC-1β expression, but more significant between-group differences in PGC-1α expression.

Previous studies have suggested that VO_2max_ and aerobic capacity are not only limited by cardiorespiratory factors, but also by the mitochondria^33,38^. In line with this theory, our correlation analyses showed that VO_2max_ was positively correlated with both PGC-1α and PGC-1β expression in some cell types in elderly women, while in young women VO_2max_ was only correlated with MNC PGC-1α expression. A positive association between PGC-1α expression and VO_2max_ has also previously been found in lifelong trained male football players^37^. Due to the noticeably higher VO_2max_ in YF compared to YC, a higher PGC-1α expression was expected in YF. However, VO_2max_ varied a lot within YF (33–55 ml/min/kg) and was markedly lower compared to other young female top athletes, e.g. middle-distance runners with VO_2max_ values of ~ 55 ml/min/kg^41^. It could be speculated that a lower variation within YF, or a specific lower limit regarding VO_2max_, would have affected our findings. Our correlation analyses also showed that body fat percentage and A/G ratio were negatively correlated with PGC-1α and PGC-1β expression in elderly women, which is in line with previous findings in women of various ages^30,31^. The demonstrated correlations emphasise the importance of preserving a reasonable physical fitness (VO_2max_) through regular participation in aerobic exercise, while avoiding too much body fat, to achieve healthy aging.

Several hallmarks of aging have been identified^2^, but neither of the aging theories appear to be fully satisfactory, and the complex interconnections are still a major challenge in aging research. Telomere length and mitochondrial function both exhibit a profound impact on the aging process, and pathological dysfunction in either has been proven to accelerate aging^2^. Thus, a direct association between telomere shortening and mitochondrial dysfunction has been proposed. During repetitive cell division, the absence or insufficient activity of telomerase results in telomere attrition, loss of chromosome ‘capping’ function and activation of p53^7^. In general, p53 is postulated to mediate growth arrest, senescence, and apoptosis in tissues with a high turnover. p53 has, however, also been shown to decrease mitochondrial function through binding and suppression of PGC-1α and PGC-1β and their downstream gene network^8^. This telomere–p53–PGC axis is argued to compromise metabolism and organ function and contribute to development of age-related disorders. In the present study, telomere length and mtDNA copy number were positively correlated in all cell types in the elderly women indicating a potential connection between the two. Existence of a telomere-mitochondria interplay in leukocytes has previously been found in different groups of participants^42^, and a co-regulation due to oxidative stress, inflammation and repeated cell replication is possible^5,42^. It might be speculated that the beneficial effects of football and team handball training in the present study were a result of reduced oxidative stress and inflammation, but more studies are required to establish this.

Cellular senescence and cell death are other central hallmarks of aging associated with telomere shortening and mitochondrial dysfunction^2^. Due to a weaken immune system in old age, senescent cells are accumulated in all types of body tissue. This accumulation reduces tissue repair and increases chronic inflammation, which affects the progression of aging and increases the risk of age-related diseases^43^. Hence, telomere shortening and mitochondrial dysfunction are linked to the onset and progression of many of the same age-related diseases, such as cardiovascular and neurodegenerative diseases as well as metabolic disorders like type 2 diabetes^5,44^. PGC-1α seems to play a key role in endothelial cell regulation and atherosclerosis, as an upregulation of the gene has been shown to prevent development of, or even reduce, atherosclerotic lesions^45^. Thus, the finding of a higher expression of key regulators of mitochondrial biogenesis and function in EH may indicate a significant reduced risk of CVD. Furthermore, numerous studies have shown that mitochondrial dysfunction is associated with muscle wasting in different muscular disorders, and that elevated PGC-1α levels may postpone the onset and reduce the progression of age-related loss of muscle mass^44^. EH demonstrated an impressive total lean mass that was 3 kg higher than observed in YC despite an age difference of more than 40 years. It is possible that the upregulated PGC-1α expression in EH was linked to muscle mass maintenance in the group despite the high age.

Finally, possessing short telomeres is associated with a mortality rate that is almost twice as high as in those having longer telomeres^4^. Interestingly, telomere length may predict mortality risk in young individuals in particular, as the mortality association diminishes with age^46^. Hence, the noticeably longer telomeres in YF may result in a markedly reduced mortality risk. Whether telomere attrition and mitochondrial dysfunction are causative or secondary effects of the diseases are not fully established, and more research within this aging topic is needed.

As with all cross-sectional studies, some degree of self-selection or confounding cannot be ruled out. It is plausible that the exercise groups in general have a healthier lifestyle than the sedentary groups, which most likely poses an additive effect of the sports participation itself on the measured aging markers. Indeed, telomere length is influenced by several other factors, such as dietary and smoking habits, perceived stress levels, socioeconomic status, chronic inflammation and paternal age^4^. To exclude the effects of confounding factors and natural genetics, a large randomised controlled trial is needed. As oestrogen is assumed to play a role in telomere regulation, measurement of circulating oestradiol and inclusion of this in the correlation analysis could have been interesting. Although the effect of both team handball and football training was investigated in the present study, a direct comparison of the two exercise types was not attempted. Similar team sports, such as basketball or floorball, may have a comparable effect on aging, although this has not yet been investigated.

In summary, this cross-sectional study showed that elite football and lifelong team handball training are associated with beneficial anti-aging cellular effects in MNCs in women. Specifically, young elite football players demonstrated higher telomere length and higher mtDNA copy number compared to young untrained controls, while elderly team handball players showed higher PGC-1α and PGC-1β expression compared to elderly untrained controls. These cellular adaptations were positively correlated with VO_2max_ and the amount of weekly exercise in MNCs and lymphocytes, emphasising the importance of preserving a reasonable fitness and activity level irrespective of age. As telomere shortening and mitochondrial dysfunction are highly associated with several age-related diseases and mortality, our findings indicate that women engaged in team sports such as football and team handball may potentially increase their health span and, ultimately, lifespan.

## Methods

### Participants

This study comprised a relatively large sample size of 129 healthy (no chronic diseases) and non-smoking (> 1 year) women. The sample size was based on previous studies of similar character conducted by the group of authors^24^ and former collaborators^23^. During the recruiting process, a total of 290 women were screened for participation, of whom 161 were excluded due to lack of compliance with the inclusion criteria (*n* = 84) or because they declined to participate after consideration (*n* = 77). The included participants were allocated into one of four groups: young elite football players (YF) aged 18–30 years (22.5 ± 0.6 years, *n* = 29); untrained young controls (YC) aged 18–30 years (24.9 ± 0.4 years, *n* = 30); lifelong trained elderly team handball players (EH) aged 60–80 years (63.9 ± 0.7 years, *n* = 35); and untrained elderly controls (EC) aged 60–80 years (66.1 ± 0.6 years, *n* = 35). EH were recruited from team handball clubs all over Denmark with help from the Danish Handball Federation (Dansk Håndbold Forbund, DHF), whereas YF were recruited from football teams in Zealand, Denmark, competing in one of the two best female leagues in Denmark (3F League or 1^st^ Division). The age-matched untrained controls were recruited through local newspapers, local institutions, and online advertisements.

YF had a history of 14.9 ± 0.6 years of regular football training and had been playing at a high level for 6.2 ± 0.6 years. On average, their age of debut was 6.9 ± 0.5 years. YF had a total of 6.7 ± 0.2 h of football training per week, including 1.0 ± 0.0 90-min matches, plus 2.3 ± 0.4 h of other types of training per week, primarily resistance training and running. Including all types of exercise, YF had been regularly physically active for 17.7 ± 0.7 years at the time of the study. EH had a history of 43.3 ± 2.0 years of regular team handball training and their age of debut was 12.1 ± 1.4 years. At the time of the study, EF had a total of 2.0 ± 0.1 h of team handball training per week, including 0.6 ± 0.1 50-min matches. Besides team handball training, EH had 2.7 ± 0.4 h of other types of training per week, e.g. jogging, resistance training, cycling, yoga, swimming, dancing and gymnastics. Including all types of exercise, EH had been regularly physically active for 50.2 ± 1.6 years. YC and EC had not participated in regular physical exercise for 4.8 ± 0.9 and 14.7 ± 3.2 years, respectively, or had never participated in a regular exercise programme (*n* = 10 in EC). Furthermore, the untrained controls had never engaged in sport at a high level.

Among the young women, the use of hormonal contraception included birth control pills (*n* = 15 in YF, *n* = 12 in YC) and an intrauterine device (*n* = 2 in YF, *n* = 3 in YC). None of the elderly women took hormone supplements due to menopause, but some used blood-pressure-lowering (*n* = 2 in EH, *n* = 9 in EC) or cholesterol-lowering (*n* = 2 in EH, *n* = 6 in EC) medication due to mild-to-moderate hypertension or hyperlipidaemia. None of the participants had been exposed to chemotherapy or radiation therapy. All subjects provided written informed consent. The study was carried out in accordance with the Declaration of Helsinki and approved by the local ethical committee of the Capital Region of Denmark (journal. no. H-15009312).

### Clinical testing

All clinical testing was carried out in the morning after an overnight fast (> 8 h). The effect of the menstrual cycle and associated hormonal fluctuations in the young women was considered carefully by consistent sampling of the blood within a narrow timespan according to each participant’s individual menstrual cycle (days 3–9). The participants were not allowed to perform any strenuous exercise for 48 h prior to testing to exclude acute effects. A peripheral venous blood sample was collected in resting state and under standardised conditions. 60 ml of sodium citrate blood was used for isolation of mononuclear cells (MNCs), as described below. Prior to blood sampling, whole-body dual-energy X-ray absorptiometry (DXA) was performed to evaluate body fat percentage, fat distribution and lean body mass. The effective radiation dose for the DXA scan was 4.66 μSv, and all analyses were performed using enCORE Version 14.10 software (GE Healthcare). On a separate test day (> 48 h from the first test day), maximal oxygen consumption (VO_2max_) was measured during a maximal fitness test on an ergometer bike using a computerised metabolic measurement system (Oxycon Pro®). The young participants completed two submaximal loads (40 and 80 W) of 3 min each, after which the load was increased by 15 W every 30 s. The elderly participants only completed the lowest submaximal load (40 W) for 3 min, after which the load was increased by 10 W every 30 s. All participants biked until exhaustion, and VO_2max_ was calculated as the mean over 30 s when oxygen consumption peaked.

### Isolation of MNCs

Ficoll density gradient centrifugation was performed to isolate MNCs from 60 ml of sodium citrate blood, as previously described^23^, and the cell number was quantified in a Neubauer chamber after staining with Türk’s solution. Immediately after isolation, the MNCs were resuspended in a freezing medium (RPMI1640 medium + 10% foetal calf serum + 5% dimethyl sulfoxide), distributed into cryotubes and gently frozen to − 80 °C in a “Mr. Frosty” freezing container (Thermo Fisher Scientific, Braunschweig, Germany). The deep-frozen cryotubes were transported on dry ice to Dr Asghar’s lab at Karolinska Institutet in Solna (Stockholm, Sweden) and kept at − 80 °C until analysis.

### DNA and RNA isolation

Lymphocytes and monocytes were separated using the MACS cell sorting protocol (see supplementary method for details). DNA was extracted from sorted cells using a Qiagen kit (QIAamp® DNA Blood Mini Kit, cat # 52304) according to the manufacturer’s instructions. DNA was quantified using a Qubit 1 × DSDNA HS kit (cat # Q33231, Invitogen) on Qubit and diluted to 1 ng/ul for the telomere and mtDNA copy number measurement. RNA was extracted from sorted cells using a Qiagen kit (QIAamp® RNA Blood Mini Kit, cat # 990395) according to the manufacturer’s instructions. RNA was then quantified using a Qubit™ RNA HS kit (cat # Q32855, Invitrogen) on Qubit.

### Telomere and mtDNA copy number essay

Telomere length and mtDNA copy number were measured using a ScienCell kit (cat # 8958). Each 15 ul reaction contained 7.5ul QuantiNova Syber green (cat # 208054, Qiagen), 0.5 ul telomere or single-copy (SCR) or mitochondrial primers, 0.1 ul ROX (passive reference dye), 1.9 ul DNA/RNA free water and 5 ul (1 ng/ul) template DNA. For telomere quantitative polymerase chain reaction (qPCR), the thermal cycle profile comprised incubation at 50 °C for 2 min and 95 °C for 10 min before running 30 thermal cycles (95 °C for 15 s, 56 °C for 45 s and 72 °C for 45 s). For single‐copy gene and mtDNA copy number qPCR, the thermal cycle profile comprised incubation at 50 °C for 2 min and 95 °C for 10 min before running 40 thermal cycles (95 °C for 15 s, 54 °C for 45 s and 72 °C for 45 s). Each assay was run on separate plates and each plate contained a serially diluted DNA sample to calculate PCR efficiency. The PCR acceptance value was set as 100 ± 15%, and any plate producing the PCR efficiency outside this range was rerun. Each sample was run in triplicate, and mean *C*_*T*_ value was used for final calculation after carefully checking the melt curve for each sample. A reference genomic DNA was added on each plate with known telomere length (369 ± 11 kb) and mtDNA copy number (1200 ± 9 copies) per diploid cell. Δ*C*_*T*_ for both telomere length and mtDNA copy number was calculated using the formula (*C*_*T*_ target sample − *C*_*T*_ reference sample) after adjusting PCR efficiency using the Pfaffl method^47^. We then calculated ΔΔ*C*_*T*_ for both telomere length and mtDNA copy number using the formula (TELΔ*C*_*T*_ − SCRΔ*C*_*T*_* or* mtDNAΔ*C*_*T*_ − SCRΔ*C*_*T*_). Relative telomere of target sample to reference sample was calculated by 2^− ΔΔ*C*_*T*_, and the ratio was then multiplied by 369 Kb to get telomere length per diploid cell. Telomere length of diploid cell was divided by the number of chromosome ends (92) to get average telomere length at each chromosome end (2^− ΔΔ*C*_*T*_ × 369/92). mtDNA copy number per diploid cell of target sample to reference sample was calculated by 2^− ΔΔ*C*_*T*_, and the ratio was then multiplied by 1200 mtDNA copy number for each sample (2^− ΔΔ*C*_*T*_ × 1200). Our method showed very high repeatability for telomere length (ICC = 97) and mtDNA copy number (ICC = 98).

### Gene expression

cDNA was synthesised using a QuantiTec Reverse Transcriptase kit (cat # 205311) according to the manufacturer’s instructions. The plate was incubated for 10 min at 25 °C followed by 1 h at 42 °C and 5 min at 85 °C to inactivate the enzyme on a QuantStudio5 thermocycler. Relative gene expression of peroxisome proliferator-activated receptor gamma coactivator 1-alpha (PGC-1α) and beta (PGC-1β) was determined using the comparative Δ*C*_*T*_ method by calculating the *C*_*T*_ values of the target genes (PGC-1α and PGC-1β) against the *C*_*T*_ values of the reference gene (GAPDH). Both target gene and GAPDH were amplified in same wells, run in triplicate, and respective *C*_*T*_ values were averaged before performing the Δ*C*_*T*_ calculation (Δ*C*_*T*_ = *C*_*T*__Target_ − *C*_*T*__GAPDH_). Gene expression values were converted into log 2 of Δ*C*_*T*_ (2^− Δ*C*_*T*_).

PGC-1α and PGC-1β expression was measured using a TaqMan® Gene Expression Assay (cat # Hs00173304_m1, Hs00993805_m1; Applied Biosystem) on a QuantStudio 5 qPCR instrument. The total qPCR reaction of 20 µl contained 3 µl cDNA, 10 µl TaqMan® Multiplex Master Mix (cat # 4461882; Applied Biosystem), 1 µl GAPDH Assay (cat # 4485712; Applied Biosystem), 1 µl PGC-1α and PGC-1β Assay and ddH2O. The TaqMan® GAPDH Assay was added to each run as an endogenous control. The thermal profile comprised 95 °C for 20 s, followed by 45 thermal cycles (95 °C for 1 s and 60 °C for 20 s).

### Statistical analysis

Statistical analyses were performed in Stata (version 16), and figures were generated using JMP (version 14). Univariate and multivariate analyses were used to assess the differences between the groups for all the studied variables. Age was included in all multivariate models as a covariate. We used the mathematical and topological features of Spearman’s correlation (r_s_) and visualised it as arc diagrams using R-studio (Version 1.1.442) to investigate the potential correlation between variables. In the event of missing values in the dataset, these were imputed (< 5%) to complete the data set. The results remained the same with or without imputed values. Results are presented as means ± SEM and the statistical significance level was set at p < 0.05.

## Supplementary Information

Supplementary Information 1.

Supplementary Information 2.

Supplementary Information 3.

## Data Availability

Data available on request from Muhammad Asghar (asghar.muhammad@ki.se) and/or Marie Hagman (mhagman@health.sdu.dk).
