# Supplementary material for: Football and team handball training postpone cellular aging in women
Source: Sci Rep. 2021 Jun 3;11:11733. doi: 10.1038/s41598-021-91255-7 (PMC8175448; doi:10.1038/s41598-021-91255-7)
Supplement: Supplementary file 1 — Supplementary Information 1. [file 41598_2021_91255_MOESM1_ESM.pdf]

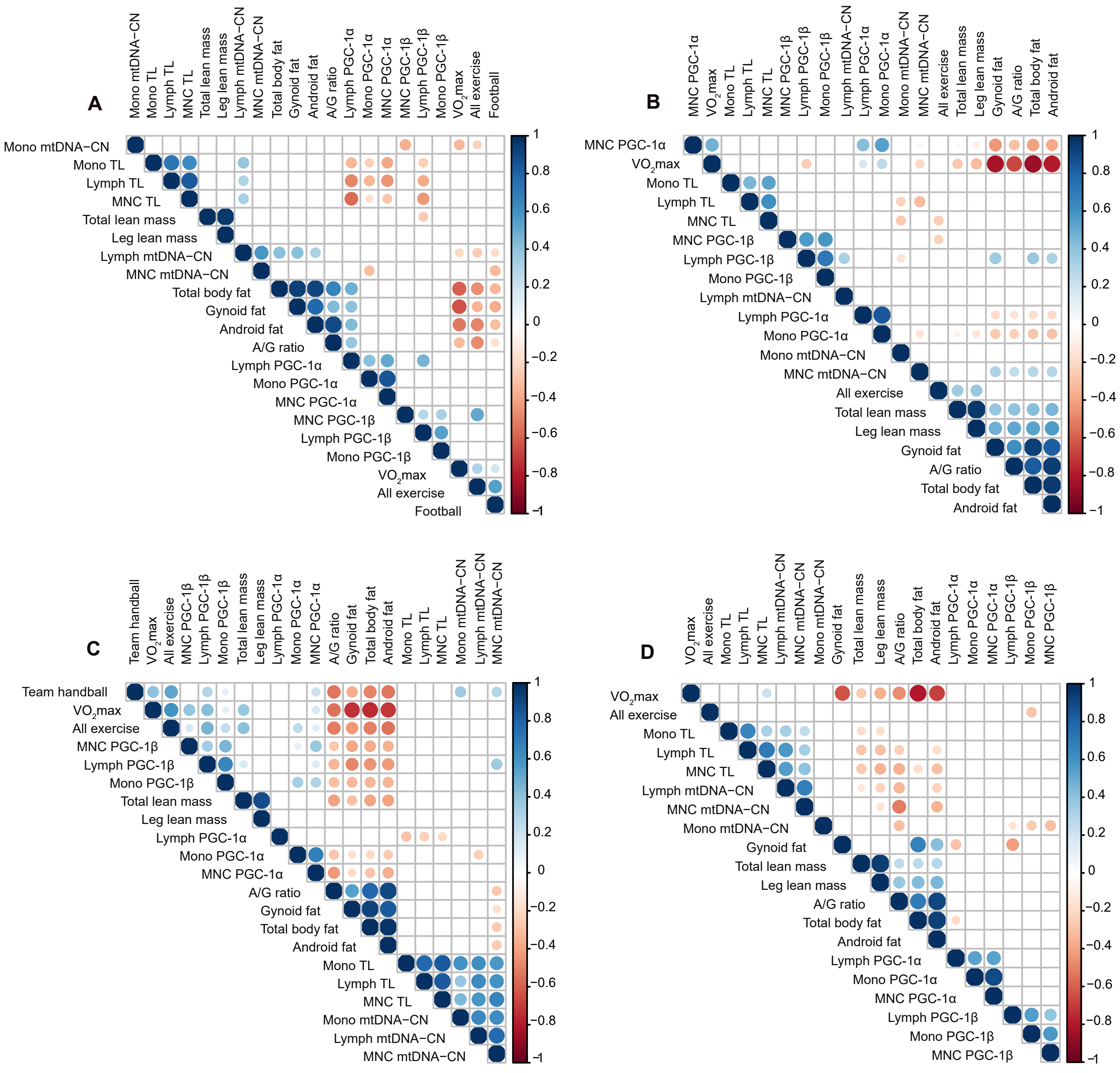
A
Mono mtDNA−CN
Mono TL
Lymph TL
MNC TL
Total lean mass
Leg lean mass
Lymph mtDNA−CN
MNC mtDNA−CN
Total body fat
Gynoid fat
Android fat
A/G ratio
Lymph PGC-1α
Mono PGC-1α
MNC PGC-1α
MNC PGC-1β
Lymph PGC-1β
Mono PGC-1β
VO2max
All exercise
Football
1
0.8
0.6
0.4
0.2
0
−0.2
−0.4
−0.6
−0.8
−1
B
MNC PGC-1α
VO2max
Mono TL
Lymph TL
MNC TL
MNC PGC-1β
Lymph PGC-1β
Mono PGC-1β
Lymph mtDNA−CN
Lymph PGC-1α
Mono PGC-1α
Mono mtDNA−CN
MNC mtDNA−CN
All exercise
Total lean mass
Leg lean mass
Gynoid fat
A/G ratio
Total body fat
Android fat
C
Team handball
VO2max
All exercise
MNC PGC-1β
Lymph PGC-1β
Mono PGC-1β
Total lean mass
Leg lean mass
Lymph PGC-1α
Mono PGC-1α
MNC PGC-1α
A/G ratio
Gynoid fat
Total body fat
Android fat
Mono TL
Lymph TL
MNC TL
Mono mtDNA−CN
Lymph mtDNA−CN
MNC mtDNA−CN
D
VO2max
All exercise
Mono TL
Lymph TL
MNC TL
Lymph mtDNA−CN
MNC mtDNA−CN
Mono mtDNA−CN
Gynoid fat
Total lean mass
Leg lean mass
A/G ratio
Total body fat
Android fat
Lymph PGC-1α
Mono PGC-1α
MNC PGC-1α
Lymph PGC-1β
Mono PGC-1β
MNC PGC-1β

Suppl. Figure 1. Spearman's correlations between the investigated variables visualised as arc diagrams in young elite football players (YF, *n*=29) **(A)**, young untrained controls (YC, *n*=30) **(B)**, elderly team handball players (EH, *n*=35) **(C)**, and elderly untrained controls (EC, *n*=35) **(D)**.
A/G ratio, android/gynoid fat ratio; All exercise, hours of weekly exercise of all types; Football, hours of weekly football training; Lymph, lymphocytes; MNC, mononuclear cells; Mono, monocytes; mtDNA-CN, mitochondrial copy number; Team handball, hours of weekly team handball training; TL, telomere length; VO_2max, maximal oxygen consumption.
