# Supplementary material for: Football and team handball training postpone cellular aging in women
Source: Sci Rep. 2021 Jun 3;11:11733. doi: 10.1038/s41598-021-91255-7 (PMC8175448; doi:10.1038/s41598-021-91255-7)
Supplement: Supplementary file 2 — Supplementary Information 2. [file 41598_2021_91255_MOESM2_ESM.pdf]

Suppl. Table 1. Univariate and multivariate analysis (ANOVA) of telomere length according to age and group in young and elderly participants.

**Telomere length**		**Univariate analysis**				**Multivariate analysis**			
Young		***Coef.***	***SE***	***t***	***p-value***	***Coef.***	***SE***	***t***	***p-value***
MNC	Age	-0.002	0.066	-0.03	0.974	0.078	0.079	1.12	0.266
	Group	-0.907	0.376	-2.41	**0.019**	-1.110	0.417	-2.66	**0.010**
Lymphocytes	Age	0.016	0.088	0.18	0.856	0.116	0.093	1.25	0.216
	Group	-1.081	0.503	-2.15	**0.036**	-1.383	0.555	-2.49	**0.016**
Monocytes	Age	0.022	0.062	0.36	0.722	0.047	0.069	0.68	0.498
	Group	-0.221	0.370	-0.60	0.552	-0.343	0.413	-0.83	0.409
Elderly									
MNC	Age	-0.032	0.028	-1.16	0.251	-0.032	0.029	-1.10	0.276
	Group	-0.077	0.230	-0.34	0.737	-0.006	0.238	-0.03	0.979
Lymphocytes	Age	-0.045	0.029	-1.54	0.127	-0.043	0.030	-1.41	0.163
	Group	-0.155	0.239	-0.65	0.520	-0.060	0.247	-0.24	0.808
Monocytes	Age	-0.006	0.028	-0.21	0.835	0.002	0.029	0.08	0.934
	Group	-0.244	0.227	-1.07	0.287	-0.249	0.238	-1.05	0.299

The multivariate analysis is corrected for age.

Suppl. Table 2. Univariate and multivariate analysis (ANOVA) of mitochondrial copy number according to age and group in young and elderly participants.

**mtDNA copy number**		**Univariate analysis**				**Multivariate analysis**			
Young		***Coef.***	***SE***	***t***	***p-value***	***Coef.***	***SE***	***t***	***p-value***
MNC	Age	-6.254	10.852	-0.58	0.567	6.680	11.478	0.58	0.563
	Group	-160.801	61.398	-2.62	**0.011**	-178.100	68.538	-2.60	**0.012**
Lymphocytes	Age	-9.290	9.321	-1.00	0.323	0.858	9.957	0.09	0.932
	Group	-137.513	53.107	-2.59	**0.012**	-139.735	59.458	-2.35	**0.022**
Monocytes	Age	-1.783	5.074	-0.35	0.727	-3.415	5.660	-0.60	0.549
	Group	13.637	30.280	0.45	0.654	22.481	33.793	0.67	0.509
Elderly									
MNC	Age	-7.889	9.242	-0.85	0.396	-11.556	9.535	-1.21	0.230
	Group	84.614	75.407	1.12	0.266	110.366	78.095	1.41	0.162
Lymphocytes	Age	-7.469	10.029	-0.74	0.459	-13.135	10.187	-1.29	0.202
	Group	141.287	80.679	1.75	0.084	170.559	83.437	2.04	**0.045**
Monocytes	Age	-2.040	3.551	-0.57	0.568	-1.135	3.695	-0.31	0.760
	Group	-29.750	28.928	-1.03	0.307	-27.219	30.264	-0.90	0.372

The multivariate analysis is corrected for age.

Suppl. Table 3. Univariate and multivariate analysis (ANOVA) of mitochondrial function according to age and group in young and elderly participants.

**Mitochondrial function**		**Univariate analysis**				**Multivariate analysis**			
Young		***Coef.***	***SE***	***t***	***p-value***	***Coef.***	***SE***	***t***	***p-value***
MNC									
PGC-1α	Age	-0.000	0.000	-1.30	0.199	-0.000	0.000	-0.66	0.511
	Group	-0.000	0.000	-1.64	0.107	-0.000	0.000	-1.18	0.242
PGC-1β	Age	-0.000	0.001	-0.26	0.798	0.000	0.001	0.26	0.795
	Group	-0.007	0.006	-1.14	0.258	-0.008	0.007	-1.14	0.261
Lymphocytes									
PGC-1α	Age	-0.000	0.000	-1.33	0.189	-0.000	0.000	-1.20	0.236
	Group	-0.000	0.000	-0.55	0.585	0.000	0.000	0.02	0.982
PGC-1β	Age	-0.000	0.001	-0.09	0.929	0.000	0.001	0.20	0.839
	Group	-0.003	0.005	-0.64	0.527	-0.004	0.006	-0.66	0.514
Monocytes									
PGC-1α	Age	-0.000	0.000	-0.32	0.751	-0.000	0.000	-0.39	0.698
	Group	0.000	0.000	0.08	0.935	0.000	0.000	0.24	0.809
PGC-1β	Age	0.000	0.000	0.87	0.390	0.000	0.001	0.39	0.700
	Group	0.003	0.003	1.20	0.235	0.003	0.003	0.91	0.369
Elderly									
MNC									
PGC-1α	Age	-0.000	0.000	-1.44	0.154	-0.000	0.000	-0.93	0.354
	Group	-0.000	0.000	-2.08	**0.041**	-0.000	0.000	-1.75	0.085
PGC-1β	Age	-0.001	0.001	-1.42	0.162	-0.001	0.001	-0.91	0.365
	Group	-0.010	0.005	-2.05	**0.044**	-0.009	0.005	-1.72	0.089
Lymphocytes									
PGC-1α	Age	-0.000	0.000	-1.34	0.186	-0.000	0.000	-0.64	0.526
	Group	-0.000	0.000	-2.87	**0.006**	-0.000	0.000	-2.57	**0.012**
PGC-1β	Age	-0.001	0.000	-1.65	0.104	-0.000	0.000	-1.22	0.228
	Group	-0.005	0.003	-1.79	0.077	-0.004	0.003	-1.40	0.166
Monocytes									
PGC-1α	Age	0.000	0.000	0.17	0.865	0.000	0.000	0.25	0.806
	Group	-0.000	0.000	-0.25	0.802	-0.000	0.000	-0.31	0.760
PGC-1β	Age	0.000	0.000	0.21	0.830	0.000	0.000	0.15	0.882
	Group	0.000	0.001	0.26	0.795	0.000	0.001	0.21	0.835

The multivariate analysis is corrected for age.
