# Supplementary material for: Football and team handball training postpone cellular aging in women
Source: Sci Rep. 2021 Jun 3;11:11733. doi: 10.1038/s41598-021-91255-7 (PMC8175448; doi:10.1038/s41598-021-91255-7)
Supplement: Supplementary file 3 — Supplementary Information 3. [file 41598_2021_91255_MOESM3_ESM.pdf]

Suppl. Table 4. Spearman's correlations between the investigated variables in all young participants (*n* =59).

	Lymph TL	Lymph mtDNA-CN	Lymph PGC-1α	Lymph PGC-1β	Mono TL	Mono mtDNA-CN	Mono PGC-1α	Mono PGC-1β	MNC TL	MNC mtDNA-CN	MNC PGC-1α	MNC PGC-1β	VO_{2max}	Total body fat	Android fat	Gynoid fat	A/G ratio	Total lean mass	Leg lean mass	All exercise	Football
Lymph TL	1	0.334	-0.234	-0.234	0.657	-0.081	-0.115	-0.068	0.797	0.117	-0.179	-0.037	0.103	-0.212	-0.180	-0.187	-0.145	0.180	0.112	0.274	0.256
Lymph mtDNA-CN		1	0.201	0.119	0.198	0.067	0.152	0.050	0.314	0.535	0.136	0.081	0.028	0.088	0.100	0.063	0.108	0.230	0.187	0.231	0.279
Lymph PGC-1α			1	0.377	-0.244	-0.003	0.646	0.021	-0.376	-0.154	0.470	0.049	0.032	0.058	0.022	0.055	0.056	0.039	0.017	0.023	0.081
Lymph PGC-1β				1	-0.210	-0.147	0.105	0.555	-0.246	0.133	0.014	0.371	0.003	0.114	0.129	0.095	0.149	-0.011	0.010	0.136	0.129
Mono TL					1	0.155	-0.126	-0.069	0.595	0.067	-0.189	-0.144	-0.090	-0.072	-0.096	-0.006	-0.152	0.130	0.087	0.062	0.079
Mono mtDNA-CN						1	0.024	0.009	-0.068	0.112	0.030	-0.206	-0.100	0.049	0.051	0.004	0.091	-0.087	-0.077	-0.101	-0.063
Mono PGC-1α							1	0.096	-0.127	-0.213	0.662	-0.061	0.114	-0.084	-0.137	-0.071	-0.119	-0.111	-0.109	-0.066	-0.019
Mono PGC-1β								1	-0.014	0.014	-0.049	0.418	-0.177	0.226	0.186	0.253	0.089	-0.042	0.059	-0.131	-0.128
MNC TL									1	0.194	-0.070	-0.003	0.163	-0.173	-0.134	-0.134	-0.130	0.137	0.100	0.302	0.287
MNC mtDNA-CN										1	-0.057	0.157	0.117	0.022	0.058	0.029	0.077	0.201	0.187	0.244	0.251
MNC PGC-1α											1	0.079	0.339	-0.196	-0.239	-0.198	-0.189	0.034	0.015	0.158	0.211
MNC PGC-1β												1	0.188	-0.127	-0.131	-0.116	-0.133	-0.016	-0.016	0.268	0.161
VO_{2max}													1	-0.825	-0.749	-0.839	-0.565	0.191	0.114	0.687	0.679
Total body fat														1	0.953	0.952	0.782	-0.076	0.020	-0.603	-0.586
Android fat															1	0.846	0.923	-0.012	0.086	-0.522	-0.497
Gynoid fat																1	0.595	-0.114	-0.021	-0.635	-0.638
A/G ratio																	1	0.039	0.121	-0.374	-0.323
Total lean mass																		1	0.971	0.463	0.520
Leg lean mass																			1	0.399	0.457
All exercise																				1	0.957
Football																					1

A/G ratio, android/gynoid fat ratio; All exercise, hours of weekly exercise of all types; Football, hours of weekly football training; Lymph, lymphocytes; MNC, mononuclear cells; Mono, monocytes; mtDNA-CN, mitochondrial copy number; TL, telomere length; VO_{2max}, maximal oxygen consumption.

Suppl. Table 5. Spearman's correlations between the investigated variables in all elderly participants (*n* =70).

	Lymph TL	Lymph mtDNA-CN	Lymph PGC-1α	Lymph PGC-1β	Mono TL	Mono mtDNA-CN	Mono PGC-1α	Mono PGC-1β	MNC TL	MNC mtDNA-CN	MNC PGC-1α	MNC PGC-1β	VO_{2max}	Total body fat	Android fat	Gynoid fat	A/G ratio	Total lean mass	Leg lean mass	All exercise	Team handball
Lymph TL	1	0.556	-0.157	0.184	0.732	0.323	-0.090	0.148	0.770	0.454	-0.032	0.059	0.109	-0.182	-0.194	-0.106	-0.219	-0.135	-0.177	-0.030	0.084
Lymph mtDNA-CN		1	0.075	-0.005	0.413	0.337	-0.089	-0.046	0.541	0.724	-0.103	-0.094	-0.107	0.056	-0.038	0.193	-0.163	-0.159	-0.176	-0.194	-0.157
Lymph PGC-1α			1	0.347	-0.203	0.192	0.298	0.031	-0.116	0.112	0.329	-0.028	0.201	-0.089	-0.054	-0.167	0.058	0.123	0.120	0.273	0.217
Lymph PGC-1β				1	0.050	0.124	0.034	0.527	0.184	0.156	0.165	0.376	0.419	-0.413	-0.359	-0.490	-0.225	0.211	0.157	0.418	0.292
Mono TL					1	0.471	-0.098	0.031	0.598	0.400	-0.038	-0.105	0.050	-0.162	-0.176	-0.095	-0.188	-0.085	-0.146	-0.016	0.192
Mono mtDNA-CN						1	-0.021	-0.186	0.218	0.383	0.012	-0.260	0.100	-0.146	-0.163	-0.047	-0.192	0.000	-0.019	0.144	0.228
Mono PGC-1α							1	0.228	-0.021	0.164	0.740	0.020	0.002	-0.054	-0.088	-0.034	-0.107	0.037	0.004	0.077	-0.007
Mono PGC-1β								1	0.119	0.110	0.190	0.451	-0.037	-0.064	-0.077	-0.160	-0.045	0.095	0.052	0.029	-0.030
MNC TL									1	0.512	0.018	-0.025	0.089	-0.220	-0.269	-0.110	-0.313	-0.124	-0.204	-0.029	0.097
MNC mtDNA-CN										1	0.125	-0.039	0.004	-0.106	-0.221	0.087	-0.345	0.012	-0.051	-0.035	-0.039
MNC PGC-1α											1	0.302	0.211	-0.285	-0.323	-0.225	-0.322	0.099	0.019	0.217	0.262
MNC PGC-1β												1	0.304	-0.291	-0.262	-0.359	-0.165	0.108	-0.003	0.249	0.223
VO_{2max}													1	-0.812	-0.742	-0.771	-0.538	0.301	0.118	0.675	0.590
Total body fat														1	0.959	0.879	0.759	-0.260	0.005	-0.582	-0.555
Android fat															1	0.741	0.903	-0.213	0.045	-0.546	-0.500
Gynoid fat																1	0.397	-0.271	-0.037	-0.560	-0.537
A/G ratio																	1	-0.151	0.049	-0.426	-0.372
Total lean mass																		1	0.926	0.482	0.400
Leg lean mass																			1	0.369	0.276
All exercise																				1	0.830
Team handball																					1

A/G ratio, android/gynoid fat ratio; All exercise, hours of weekly exercise of all types; Lymph, lymphocytes; MNC, mononuclear cells; Mono, monocytes; mtDNA-CN, mitochondrial copy number; Team handball, hours of weekly team handball training; TL, telomere length; VO_{2max}, maximal oxygen consumption.

Suppl. Table 6. Spearman's correlations between the investigated variables in young elite football players (YF, *n* =29).

	Lymph TL	Lymph mtDNA-CN	Lymph PGC-1α	Lymph PGC-1β	Mono TL	Mono mtDNA-CN	Mono PGC-1α	Mono PGC-1β	MNC TL	MNC mtDNA-CN	MNC PGC-1α	MNC PGC-1β	VO_{2max}	Total body fat	Android fat	Gynoid fat	A/G ratio	Total lean mass	Leg lean mass	All exercise	Football
Lymph TL	1	0.307	-0.489	-0.377	0.727	0.016	-0.337	-0.096	0.836	0.199	-0.442	-0.100	-0.138	-0.119	-0.081	-0.059	-0.097	0.025	-0.017	0.066	-0.065
Lymph mtDNA-CN		1	-0.078	-0.020	0.381	0.206	-0.071	-0.034	0.321	0.584	0.072	-0.065	-0.209	0.420	0.313	0.420	0.156	0.053	0.003	-0.252	-0.188
Lymph PGC-1α			1	0.468	-0.337	-0.077	0.414	0.055	-0.562	-0.195	0.514	0.017	-0.149	0.488	0.434	0.407	0.382	0.010	0.033	-0.057	0.250
Lymph PGC-1β				1	-0.242	-0.144	0.046	0.546	-0.437	0.116	0.063	0.281	0.011	0.098	0.138	0.096	0.178	-0.260	-0.239	0.196	0.251
Mono TL					1	0.278	-0.233	-0.053	0.627	0.140	-0.380	-0.163	-0.274	-0.001	-0.056	0.096	-0.161	0.216	0.158	-0.030	0.008
Mono mtDNA-CN						1	0.158	0.111	0.072	0.007	-0.025	-0.360	-0.325	0.134	0.132	0.079	0.128	-0.058	-0.050	-0.221	-0.073
Mono PGC-1α							1	0.122	-0.172	-0.303	0.856	-0.230	0.068	0.161	0.058	0.103	0.043	-0.167	-0.079	-0.208	-0.006
Mono PGC-1β								1	-0.142	0.046	-0.048	0.329	-0.115	-0.008	-0.015	0.021	-0.011	-0.165	-0.063	-0.010	0.017
MNC TL									1	0.132	-0.270	-0.097	-0.086	-0.031	0.018	0.028	-0.042	-0.033	-0.058	0.076	-0.061
MNC mtDNA-CN										1	-0.185	0.220	-0.065	0.232	0.224	0.274	0.144	0.127	0.079	-0.181	-0.327
MNC PGC-1α											1	-0.075	0.105	0.289	0.131	0.237	0.056	-0.125	-0.085	-0.131	0.059
MNC PGC-1β												1	0.063	0.001	-0.077	0.073	-0.162	-0.114	-0.125	0.515	0.115
VO_{2max}													1	-0.594	-0.520	-0.636	-0.319	-0.201	-0.251	0.305	0.198
Total body fat														1	0.918	0.940	0.664	0.117	0.151	-0.463	-0.330
Android fat															1	0.789	0.891	0.067	0.103	-0.491	-0.306
Gynoid fat																1	0.437	0.170	0.221	-0.335	-0.364
A/G ratio																	1	0.001	0.021	-0.474	-0.177
Total lean mass																		1	0.964	-0.095	0.207
Leg lean mass																			1	-0.148	0.136
All exercise																				1	0.545
Football																					1

A/G ratio, android/gynoid fat ratio; All exercise, hours of weekly exercise of all types; Football, hours of weekly football training; Lymph, lymphocytes; MNC, mononuclear cells; Mono, monocytes; mtDNA-CN, mitochondrial copy number; TL, telomere length; VO_{2max}, maximal oxygen consumption.

Suppl. Table 7. Spearman's correlations between the investigated variables in young untrained controls (YC, *n* =30).

	Lymph TL	Lymph mtDNA-CN	Lymph PGC-1α	Lymph PGC-1β	Mono TL	Mono mtDNA-CN	Mono PGC-1α	Mono PGC-1β	MNC TL	MNC mtDNA-CN	MNC PGC-1α	MNC PGC-1β	VO_{2max}	Total body fat	Android fat	Gynoid fat	A/G ratio	Total lean mass	Leg lean mass	All exercise
Lymph TL	1	0.231	0.143	0.082	0.457	-0.227	0.187	0.056	0.614	-0.317	0.110	-0.040	-0.076	-0.027	-0.051	0.026	-0.041	0.098	-0.008	-0.116
Lymph mtDNA-CN		1	0.492	0.329	-0.166	-0.031	0.368	0.215	0.123	0.362	0.083	0.173	-0.316	0.289	0.307	0.257	0.288	0.123	0.108	-0.154
Lymph PGC-1α			1	0.244	-0.115	0.076	0.849	0.004	-0.183	-0.155	0.424	0.078	0.167	-0.180	-0.192	-0.189	-0.151	0.029	-0.040	-0.159
Lymph PGC-1β				1	-0.145	-0.158	0.217	0.713	0.150	0.110	-0.134	0.567	-0.241	0.371	0.315	0.363	0.237	0.273	0.326	-0.115
Mono TL					1	-0.025	0.005	-0.077	0.531	-0.153	0.099	-0.152	-0.058	-0.082	-0.098	-0.011	-0.131	-0.076	-0.107	-0.160
Mono mtDNA-CN						1	-0.082	-0.089	-0.259	0.289	0.113	-0.008	0.161	-0.054	-0.032	-0.143	0.046	-0.080	-0.067	0.084
Mono PGC-1α							1	0.075	-0.087	-0.143	0.541	0.112	0.261	-0.274	-0.281	-0.262	-0.233	-0.079	-0.141	-0.119
Mono PGC-1β								1	0.263	0.072	0.002	0.588	-0.124	0.302	0.225	0.377	0.089	0.190	0.297	-0.129
MNC TL									1	0.070	0.079	0.043	-0.013	0.033	0.011	0.135	-0.045	-0.001	-0.023	-0.248
MNC mtDNA-CN										1	-0.058	-0.035	-0.208	0.282	0.288	0.309	0.243	-0.031	0.033	0.003
MNC PGC-1α											1	0.225	0.478	-0.409	-0.376	-0.436	-0.290	-0.038	-0.092	-0.104
MNC PGC-1β												1	0.206	-0.111	-0.073	-0.165	-0.039	-0.096	-0.052	-0.221
VO_{2max}													1	-0.856	-0.787	-0.830	-0.669	-0.266	-0.317	-0.011
Total body fat														1	0.956	0.937	0.831	0.425	0.533	0.126
Android fat															1	0.826	0.948	0.459	0.568	0.177
Gynoid fat																1	0.617	0.388	0.482	0.019
A/G ratio																	1	0.410	0.512	0.243
Total lean mass																		1	0.966	0.359
Leg lean mass																			1	0.371
All exercise																				1

A/G ratio, android/gynoid fat ratio; All exercise, hours of weekly exercise of all types; Lymph, lymphocytes; MNC, mononuclear cells; Mono, monocytes; mtDNA-CN, mitochondrial copy number; TL, telomere length; VO_{2max}, maximal oxygen consumption.

Suppl. Table 8. Spearman's correlations between the investigated variables in elderly team handball players (EH, *n* =35).

	Lymph TL	Lymph mtDNA-CN	Lymph PGC-1α	Lymph PGC-1β	Mono TL	Mono mtDNA-CN	Mono PGC-1α	Mono PGC-1β	MNC TL	MNC mtDNA-CN	MNC PGC-1α	MNC PGC-1β	VO_{2max}	Total body fat	Android fat	Gynoid fat	A/G ratio	Total lean mass	Leg lean mass	All exercise	Team handball
Lymph TL	1	0.638	-0.243	0.230	0.782	0.387	-0.073	0.314	0.839	0.608	-0.090	0.070	0.056	-0.200	-0.172	-0.158	-0.182	-0.101	-0.143	-0.141	0.041
Lymph mtDNA-CN		1	0.096	0.114	0.615	0.642	-0.241	-0.072	0.584	0.773	-0.156	-0.230	0.038	-0.002	-0.028	0.064	-0.092	-0.005	0.078	-0.066	0.127
Lymph PGC-1α			1	0.331	-0.289	0.176	0.303	0.076	-0.207	0.167	0.255	-0.105	0.083	0.069	0.080	-0.020	0.172	0.051	0.100	0.157	0.007
Lymph PGC-1β				1	0.066	0.195	0.063	0.653	0.313	0.360	0.150	0.343	0.426	-0.449	-0.428	-0.482	-0.338	0.168	0.133	0.468	0.291
Mono TL					1	0.607	-0.168	0.130	0.831	0.577	-0.132	-0.125	-0.032	-0.197	-0.194	-0.134	-0.199	-0.137	-0.236	-0.211	0.214
Mono mtDNA-CN						1	-0.170	-0.108	0.446	0.629	-0.147	-0.297	0.120	-0.095	-0.091	-0.079	-0.066	-0.051	-0.083	0.088	0.354
Mono PGC-1α							1	0.337	-0.029	-0.057	0.685	0.115	0.135	-0.207	-0.256	-0.160	-0.273	0.093	-0.030	0.257	0.052
Mono PGC-1β								1	0.271	0.071	0.302	0.454	0.111	-0.340	-0.335	-0.323	-0.302	0.026	-0.069	0.243	0.107
MNC TL									1	0.668	-0.042	-0.053	-0.007	-0.262	-0.254	-0.201	-0.271	-0.013	-0.081	-0.108	0.201
MNC mtDNA-CN										1	0.017	-0.158	0.204	-0.252	-0.258	-0.173	-0.262	0.193	0.200	0.166	0.292
MNC PGC-1α											1	0.387	0.173	-0.289	-0.368	-0.188	-0.422	0.068	-0.031	0.125	0.215
MNC PGC-1β												1	0.396	-0.376	-0.343	-0.383	-0.263	-0.023	-0.155	0.169	0.085
VO_{2max}													1	-0.748	-0.707	-0.728	-0.542	0.382	0.153	0.595	0.401
Total body fat														1	0.971	0.930	0.795	-0.394	-0.035	-0.501	-0.492
Android fat															1	0.834	0.910	-0.403	-0.067	-0.542	-0.540
Gynoid fat																1	0.544	-0.294	0.038	-0.437	-0.364
A/G ratio																	1	-0.405	-0.151	-0.513	-0.536
Total lean mass																		1	0.883	0.411	0.100
Leg lean mass																			1	0.291	-0.080
All exercise																				1	0.522
Team handball																					1

A/G ratio, android/gynoid fat ratio; All exercise, hours of weekly exercise of all types; Lymph, lymphocytes; MNC, mononuclear cells; Mono, monocytes; mtDNA-CN, mitochondrial copy number; Team handball, hours of weekly team handball training; TL, telomere length; VO_{2max}, maximal oxygen consumption.

Suppl. Table 9. Spearman's correlations between the investigated variables in elderly untrained controls (EC, *n* =35).

	Lymph TL	Lymph mtDNA-CN	Lymph PGC-1α	Lymph PGC-1β	Mono TL	Mono mtDNA-CN	Mono PGC-1α	Mono PGC-1β	MNC TL	MNC mtDNA-CN	MNC PGC-1α	MNC PGC-1β	VO_{2max}	Total body fat	Android fat	Gynoid fat	A/G ratio	Total lean mass	Leg lean mass	All exercise
Lymph TL	1	0.575	-0.028	0.040	0.652	0.218	-0.107	-0.005	0.706	0.334	0.014	-0.010	0.128	-0.107	-0.187	0.064	-0.240	-0.275	-0.292	-0.313
Lymph mtDNA-CN		1	0.310	-0.083	0.337	0.163	-0.009	-0.055	0.554	0.688	0.026	0.163	-0.043	-0.088	-0.225	0.178	-0.332	-0.128	-0.229	-0.117
Lymph PGC-1α			1	0.201	-0.104	0.207	0.538	0.006	0.017	0.190	0.539	0.025	0.164	-0.199	-0.123	-0.288	-0.008	-0.017	-0.057	-0.018
Lymph PGC-1β				1	-0.119	-0.146	0.006	0.553	-0.067	-0.126	0.079	0.398	0.214	-0.141	-0.013	-0.418	0.160	0.155	0.077	-0.119
Mono TL					1	0.225	-0.017	-0.067	0.320	0.256	0.058	-0.165	0.009	0.041	-0.042	0.164	-0.115	-0.185	-0.180	-0.075
Mono mtDNA-CN						1	0.131	-0.257	-0.036	0.178	0.195	-0.305	-0.103	-0.116	-0.182	0.177	-0.302	-0.065	-0.048	0.111
Mono PGC-1α							1	0.148	-0.014	0.327	0.896	-0.097	-0.140	0.083	0.052	0.081	0.024	0.023	0.040	-0.139
Mono PGC-1β								1	0.003	0.127	0.111	0.580	-0.162	0.179	0.135	-0.096	0.161	0.217	0.181	-0.278
MNC TL									1	0.401	0.075	-0.010	0.217	-0.187	-0.303	0.029	-0.354	-0.270	-0.343	-0.179
MNC mtDNA-CN										1	0.338	0.217	-0.072	-0.116	-0.343	0.303	-0.518	-0.013	-0.153	-0.014
MNC PGC-1α											1	0.003	0.017	-0.086	-0.107	-0.065	-0.096	-0.049	-0.083	-0.077
MNC PGC-1β												1	-0.221	0.144	0.097	-0.081	0.129	0.089	0.009	-0.092
VO_{2max}													1	-0.790	-0.683	-0.633	-0.463	-0.252	-0.351	0.128
Total body fat														1	0.931	0.672	0.709	0.269	0.436	-0.207
Android fat															1	0.428	0.903	0.287	0.454	-0.256
Gynoid fat																1	0.009	0.159	0.281	0.034
A/G ratio																	1	0.254	0.383	-0.269
Total lean mass																		1	0.944	0.129
Leg lean mass																			1	0.118
All exercise																				1

A/G ratio, android/gynoid fat ratio; All exercise, hours of weekly exercise of all types; Lymph, lymphocytes; MNC, mononuclear cells; Mono, monocytes; mtDNA-CN, mitochondrial copy number; TL, telomere length; VO_{2max}, maximal oxygen consumption.
